# Influence of Anthropometry, Age, Sex, and Activity Level on the Hand Reach Star Excursion Balance Test

**DOI:** 10.3389/fpsyg.2019.00756

**Published:** 2019-04-09

**Authors:** Ola Eriksrud, Peter A. Federolf, Jan Cabri

**Affiliations:** ^1^Department of Physical Performance, Norwegian School of Sports of Science, Oslo, Norway; ^2^Department of Sport Science, University of Innsbruck, Innsbruck, Austria

**Keywords:** mobility, postural control, testing, instrument development, normalization

## Abstract

The influence of anthropometric measurements, age, sex, and activity level have been found to influence tests of dynamic postural control such as the star excursion balance test (SEBT). The hand reach star excursion balance test (HSEBT) measures different aspects of dynamic postural control. The purpose of the present study was to explore the influence of these factors on the HSEBT. A convenience sample of 223 subjects performed four horizontal (L45, R45, L135, and R135) and two rotational (LROT and RROT) reaches. The influence of anthropometric measurements (height, arm length, leg length, and wingspan) on reach measurements were assessed using stepwise multiple linear regression. Influence of age (young: <20 years; adult: >20 years), sex (male; female) and activity level (athletes; recreational) on reach measurements were analyzed using independent samples *t*-test (*p* < 0.05) and interpreted using effect size (Cohens *d*) and established values of minimal detectable change (MDC). Wingspan explained a significant portion of the variance of only R45 (34.6%) and L45 (11.7%) reach measurements and normalized (percentage of wingspan). A medium effect of age, sex, and activity level was observed for normalized L45 and R45 reaches (*d* = 0.50–72). Group differences greater than MDC values and a medium effect for age (*d* = 0.55) and activity level (*d* = 0.75) were observed for the R135 reach. L45 and R45 reaches should be normalized to wingspan, but not the other reaches. Between individual or group comparisons should consider age, activity level and sex as potential covariates.

## Introduction

The hand reach star excursion balance test (HSEBT) has proven to be a valid and reliable measurement tool for dynamic postural control (Eriksrud et al., 2017). The hand reaches performed on each foot capture different aspects of dynamic postural control as compared to the well-established star excursion balance test (SEBT) (Eriksrud et al., 2018). Furthermore, it measures functional mobility, i.e., the combined utilization of the ranges of motion (ROMs) of multiple joints for the accomplishment of activities of daily living and athletic performance in an ecological manner. In comparison to the SEBT the HSEBT elicits greater lower extremity and trunk movements with additional hip (extension) and upper extremity joint movements. Specifically, when compared to conventional ROM data, 8 of 22 joint movements were within these normative ranges (Eriksrud et al., 2018).

Currently, other hand reach tests such as the functional reach test (FR) (Duncan et al., 1990), standing lateral reach (Brauer et al., 1999), multidirectional reach test (Newton, 2001), and upper quarter Y balance test (Gorman et al., 2012a) are used to assess mobility and dynamic postural control. However, these tests are reaches in the horizontal plane that elicit small trunk and lower extremity joint movements (Duncan et al., 1990; Brauer et al., 1999; Newton, 2001), or are performed in positions non-specific to standing (i.e., planked position) (Gorman et al., 2012a). Since many actions in sports and activities of daily living are based on hand interactions with the environment (e.g., pushing, pulling, reaching, throwing) the HSEBT represents an alternative assessment offering better specificity in relation to such tasks (Eriksrud et al., 2018).

Patients with low back pain (LBP) have an altered lumbopelvofemoral rhythm (Laird et al., 2014) commonly assessed in standing flexion movements. However, lower extremity position, width and angulation, influence this rhythm with implications on postural stability (Zhou et al., 2016). Changes in stance not only influence base of support (BOS) but also lower extremity joint movements associated with the flexion task. The HSEBT can assess the lumbopelvofemoral rhythm not only in different flexion movements, but also in extension, lateral flexion and rotational movement patterns in a standardized manner. It may provide a better measurement tool to document such a rhythm, for example, in patients with LBP.

The HSEBT also appears to be a good addition to the assessment tools used for the evaluation of risk of falling, considering that falling often occurs while reaching, leaning (Nachreiner et al., 2007) or bending (Duckham et al., 2013). The functional reach test (FR), a single item hand reach test, has been reported to predict risk of falling (Scott et al., 2007). However, falls occur in multiple directions and it might be important to assess different directions to gain information about more multifaceted boundary conditions. In fact, Newton established that horizontal reaches in the anterior-posterior and medial-lateral direction quantify different limits of stability (Newton, 2001). The HSEBT therefore represents a promising addition to the assessment tools in fall risk management considering the high similarity of some of its tests with the movements already established as risk factors (Nachreiner et al., 2007; Duckham et al., 2013).

Shoulder dysfunction and injuries are common in throwing sports (Clarsen et al., 2014). Energy contribution and transfer through the kinetic chain to the shoulder have been described (Roach and Lieberman, 2014). For example, an increased leg drive in the tennis serve has been found to be associated with smaller shoulder and elbow torques while achieving the same serve speeds (Elliott et al., 2003), thus, potentially decreasing shoulder and elbow injury risks. Furthermore, restricting mobility of the torso by bracing resulted in a significant reduction in joint power generation throughout the kinetic chain, elastic storage of energy at the shoulder, and throwing velocity (Roach and Lieberman, 2014). Considering the importance of the full kinetic chain to shoulder function, the HSEBT may be a good alternative measure for shoulder function and dysfunction.

In the comparable SEBT, outcomes are known to be influenced by anthropometry, age, activity level, and sex. Specifically, leg length was the anthropometric measurement found to explain the largest portion of the variance in the SEBT reaches (range *R*^2^:0.02–0.23). Consequently, SEBT measurements has since mostly been normalized to leg length (Gribble and Hertel, 2003; Gribble et al., 2012), while others have normalized to height (Glave et al., 2016). Physical activities influence SEBT measures, specifically, differences between sports have been observed (Bressel et al., 2007) with equivocal findings between athletes and recreational active individuals (Thorpe and Ebersole, 2008; Sabin et al., 2010; Ambegaonkar et al., 2013). The SEBT measures are also affected by sex, however there is a controversy with respect to the direction of the relationship. Sex has been found to have an equivocal effect on SEBT reach measures with no effect (Gribble and Hertel, 2003), greater reach measures in males than females (Gorman et al., 2012b; Holden et al., 2016), and vice versa (Gribble et al., 2009; Holden et al., 2016). In adolescents and young adults, the SEBT reaches were found to increase with age (Holden et al., 2016; Gonzalo-Skok et al., 2017; McCann et al., 2017).

Therefore, the purpose of the current study was to determine the influence of anthropometric measurements, age, sex, and activity level on HSEBT reaches and to provide reference values for future comparisons.

## Methods

### Participants

A convenience sample of 223 subjects participated in the study. Recreational active (*n* = 57) and handball players (*n* = 12) were recruited. We defined recreationally active as individuals that regularly participated in physical activity for at least 30 min four times a week. Furthermore, 154 athletes competing at the Youth Olympic Games (YOG) were recruited.

### Testers and Environment

Participation was voluntary and subjects were tested in different environments. The recreational active and the throwing athletes gave written informed consent prior to being tested by two experienced testers in the biomechanics laboratory of the university. The YOG athletes were evaluated at the Learn & Share area at the YOG Winter Games 2016 by four additional experienced testers (trainers and physical therapists). As a part of this experience the athletes had the opportunity to compare their HSEBT reach measurements to anonymous data from World and Olympic champions in their respective sport. The following anonymous data were obtained and stored electronically: number as an identifier without any key, anthropometry (height, leg length, wing span, and arm length), sex, sport, and year of birth. Information about the study was shown on a computer screen in English. Based on the recommendation of the International Olympic Committee this information was also available in writing in the following languages: Norwegian, Chinese, English, French, Japanese, German, Korean and Russian. Then informed consent was obtained by checking a box on the computer screen. These procedures were discussed and formulated with lawyers from the Norwegian Sports Federation, and the study was approved and authorized by the Norwegian Data Protection agency and the Regional Committees for Medical and Health Research Ethics. The study was conducted according to the Declaration of Helsinki.

### Anthropometric Measurements

Height was obtained using a Seca model 217 stadiometer (Seca GmbH. & Co. Hamburg. Germany). Leg length was measured from the greater trochanter to the floor of one leg, arm length was measured from acromion to middle digit with shoulder abducted to 90° of one arm, and wingspan from middle digit to middle digit with both shoulders abducted to 90°. These measures were done with a standard tape measure [centimeter (cm)].

### Testing Procedures

**Figure 1:**
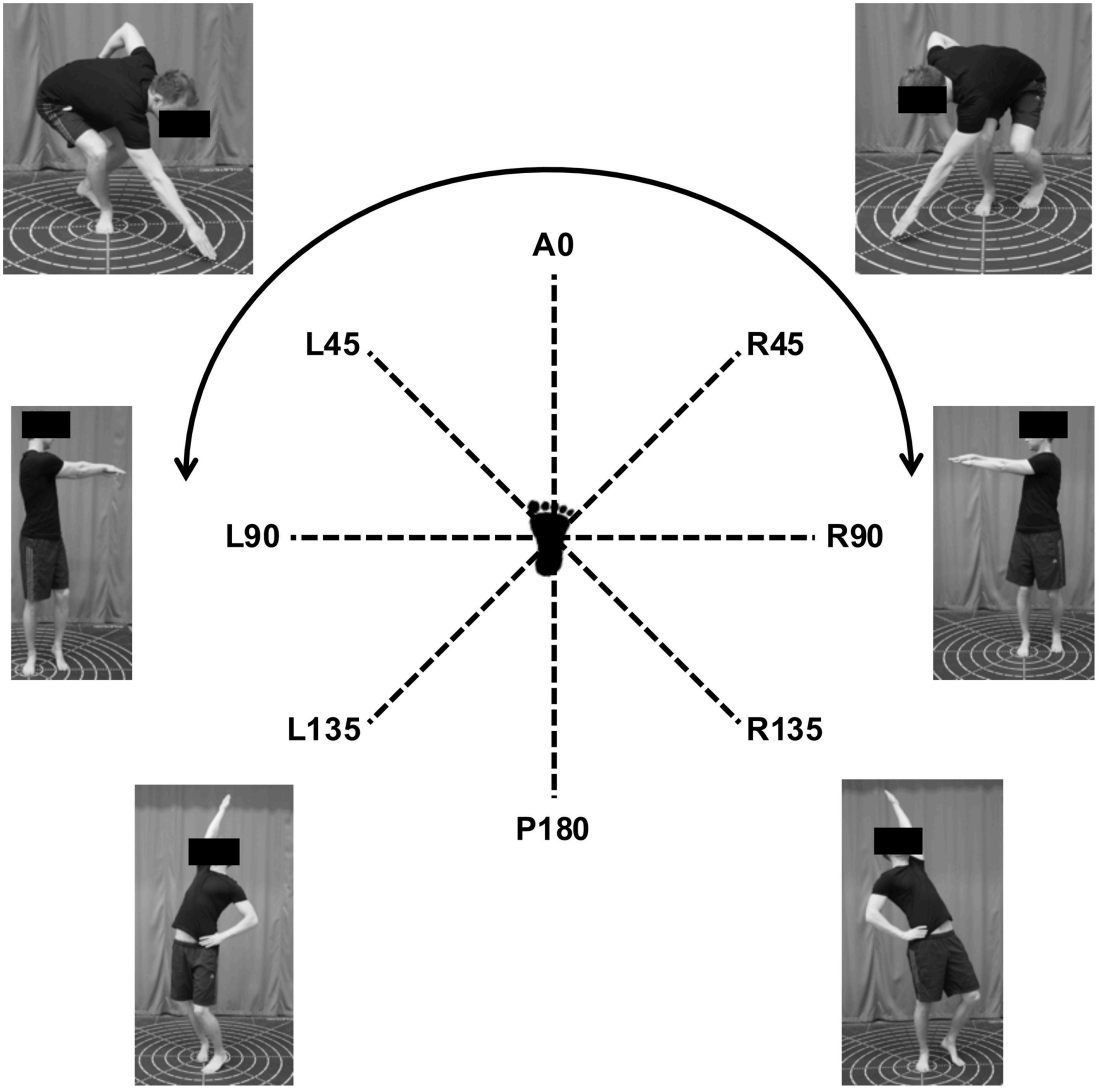
Maximum reach position of HSEBT reaches standing on the right foot.

Subjects were tested on a subset (six) of the ten hand reaches that make up the HSEBT (Eriksrud et al., 2017, 2018). For clarity, HSEBT testing procedures are summarized here. The HSEBT reaches are defined from the anatomical position where the anterior (A0) and posterior (P180) reaches divide the body into left (L) and right (R) halves. Each half is then divided into reaches at 45-degree increments (R45, R90, R135, L135, L90, and R45). Of these eight reaches the R45, R135, L135, and L45 were tested on each foot. All of these are unilateral hand reaches and the hand selected to perform the reach was based on crossing midline (line connecting the A0 and P180 reach direction) with the opposite hand placed on the hip. Reach measurements were obtained on a mat with imprinted reaching directions with marks at two cm intervals and nine concentric circles at 10 cm intervals with the outer circle (90 cm radius) marked at 10-degree intervals (Athletic Knowledge Nordic AB, Stockholm, Sweden). The foot tested (stance foot) was placed in the center of the mat while the other foot (support foot) was placed (toe touch) at a 135-degree angle relative to the reaching direction between the 20 and 30 cm concentric circle. Maximum reach measurements from the center of the testing mat to the most distal point of the middle digit was then obtained. Specifically, position of the middle digit on the testing mat (light touch and no support) (R45 and L45), and from a plumb line projecting the position of the middle digit to the testing mat (R135 and L135) were obtained. Based on sagittal plane hip joint movements at maximum reach position, the R45 and L45 are considered flexion while the R135 and the L135 are considered extension movements. In addition, both left and right rotational reaches (LROT and RROT) were measured. For the rotational reaches the stance foot is placed in the middle of the testing mat with the support foot positioned parallel between the 20 and 30 cm concentric circle and allowed to rotate in the direction of the reach. Rotational reaches are bilateral hand reaches with the middle digits on top of each other. Maximum reach position was projected onto the concentric circles and quantified as the difference from A0 (0 degrees). Pictures of maximum reach positions standing on the right foot is presented in Figure 1. For all reaches, subjects were instructed to reach or rotate as far as possible at their own rate and then return to the starting position while maintaining balance. A minimum of three practice trials were given for each test to ensure that the test was understood, after which the maximum reach of three valid test trials were recorded for analysis.

### Statistical Analysis

Descriptive statistics [mean and standard deviation (SD)] of participant characteristics (anthropometric measurements and age) and reach measurements (R45, R135, L135, R135, LROT, and RROT) shown in Figure 1 were calculated using Excel for Mac OS 10.10.5 (Apple Inc., Cupertino, CA, USA), version 14.4.8 (Microsoft Corp., Redmond, WA, USA). Mirrored reach test measurements on the left and right foot were compared using paired samples *t*-test. Side differences were interpreted based on effect size (Cohen's *d*) as follows: trivial <0.2; small 0.2–0.5; medium 0.5–0.8; large >0.8 (Cohen, 1988), and minimal detectable change (MDC) from test-retest reliability (Eriksrud et al., 2017).

The influence of anthropometric measures (height, wingspan, arm length, leg length, and trunk), age, sex, and activity level (athletes; recreational) on HSEBT measurements was determined using multiple regression analysis (IBM SPSS, v 21.0, IBM, Armonk, NY, USA). Measurements for the same tests on the left and right foot (e.g., left foot R45 reach and right foot L45 reach) were averaged. Linearity was assessed by visual inspection of scatter plots of studentized residuals and predicted values. Multicollinearity was assessed using variable inflation factor (VIF) with a cutoff of >10. Independences of residuals were analyzed using Durbin-Watson statistics with cutoff values <1 and >3. Homoscedasticity was assessed by visual inspection of the scatter plots of the standardized predicted values of the model and the standardized residuals. Normality of residuals was determined by visual inspection the histograms of standardized residuals and probability-probability plots. Casewise diagnostics were set to three standard deviations to determine if 1% or less of the subjects had standardized residuals outside this distribution. Specifically, a random sample, 75% of participants, were used to generate the initial model using forward stepwise regression based on a statistical significance (*t*-test). The model was then validated on the remaining 25% of the participants using forced entry. The validation model was then compared to the initial model based on change of *R*^2^ values, and independent variables that significantly contributed (*p* < 0.05) to the model were retained. Pearson correlation coefficients of the retained variables to their respective HSEBT reaches were then calculated. The criterion for normalization of HSEBT reaches to anthropometric measures was based on significant correlation coefficients and *R*^2^ values or changes greater than the coefficient of variation (CV) of the respective reach (Eriksrud et al., 2017).

Independent samples *t*-tests were then used to explore significant differences between age groups (young: <20 years; adult: >20 years), sex (M; F) and activity level (recreational; athletes). Homogeneity of variance was assessed using Levene's test and normal distribution was assessed using Shapiro Wilks's test. In the presence a significant Shapiro Wilk's the test z-scores of both skewness and kurtosis were calculated to explore the necessity for data transformation. Effect size was calculated using Cohen's *d* and interpreted as described above (Cohen, 1988). Outliers were removed based the criteria described by Hoaglin and Iglewics (Hoaglin and Iglewicz, 1987).

## Results

Mean values of all variables measured, of both athletic (sorted by sport) and recreational populations, are provided in Table 1. Table 2 presents mean values for age, anthropometric measures and HSEBT reach measurements organized by groups (sex, age, and activity level). In addition, significance of group differences, effect sizes and established MDC values are presented (Table 2). The male group was older than the female group (*d* = 0.83) with greater anthropometric measures (range *d* = 0.94–1.51). The adult group also had greater anthropometric measures than the young group (range *d* = 0.56–1.17). Recreational active were older than athletes (*d* = 2.00) with greater anthropometric measures (range *d* = 0.64–1.26). Females, young participants and athletes demonstrated significantly greater normalized L45 and R45 reach measurements (*p* ≤ 0.001) with medium effect sizes. Trivial effects were observed for the non-normalized comparisons for these reaches with one exception: males had greater R45 reach measurements than females (small effect) with a group difference greater than MDC values. Small to medium effects for sex, activity level and age were observed for the R135 reach. Specifically, the athletic group had reach measurements greater than MDC values, while the observed difference between the young and the adult group (7.6 cm) is within the range of MDC values. The athlete group had significantly greater L135 reach measurements than the recreational group (small effect). The observed group difference (4.1 cm) is within the range of MDC values. Trivial to small effects were observed for age, sex and activity level on rotational reach measurements (Table 2).

**Table 1:** Age, anthropometry, and HSEBT results (absolute and normalized values) of the athletic and recreational populations.

**Athletes**														**Recreational**
**Measure**	**Alpine skiing**	**Bobsleigh**	**CC and biathlon**	**Curling**	**Figure skating**	**Freestyle skiing**	**Icehockey**	**Luge**	**Skeleton**	**Snowboarding**	**Ski jumping**	**Ice skating**	**Throwing**	
Participants	28	9	4	6	14	13	36	17	8	16	1	2	12	57
Age (yrs)	17.5 ± 0.5	17.3 ± 0.5	17 ± 0	17 ± 0	16.6 ± 0.5	17.3 ± 0.6	16.6 ± 0.5	17.0 ± 0.5	17.1 ± 0.4	17.3 ± 0.6	17.0 ± 0	18.0 ± 0	21.7 ± 1.7	24.6 ± 4.9
Height (cm)	170.9 ± 4.8	170.1 ± 8.7	177.5 ± 5.4	169.8 ± 12.8	164.3 ± 7.6	170.6 ± 8.8	170.9 ± 7.9	174.6 ± 7.7	168.8 ± 5.5	167.9 ± 8.1	162.0	180.5 ± 10.6	174.8 ± 6.5	180.3 ± 7.8
Leg length (cm)	87.7 ± 3.3	87.8 ± 2.9	90.0 ± 2.7	88.3 ± 6.0	83.4 ± 5.5	88.8 ± 5.5	88.7 ± 5.8	89.5 ± 4.4	87.4 ± 3.4	85.1 ± 3.2	85.0	89.0 ± 4.2	89.7 ± 5.4	91.3 ± 5.0
Wingspan (cm)	174.8 ± 5.7	174.6 ± 12.1	178.8 ± 5.6	170.8 ± 16.2	166.6 ± 10.8	172.7 ± 9.4	172.1 ± 10.6	176.8 ± 7.7	170.5 ± 7.0	171.7 ± 9.6	167.0	186.5 ± 13.4	173.9 ± 8.6	180.2 ± 8.1
Arm length (cm)	73.5 ± 3.2	72.9 ± 5.0	73.8 ± 2.2	71.0 ± 6.8	68.9 ± 4.2	71.8 ± 3.8	72.4 ± 4.8	75.1 ± 4.7	71.3 ± 3.2	71.7 ± 40	71.0	77.5 ± 6.4	72.4 ± 4.3	75.0 ± 3.4
L SLS R45 (cm)	80.3 ± 5.6	78.0 ± 6.0	87.0 ± 2.4	76.0 ± 9.2	79.6 ± 6.2	77.4 ± 5.3	77.9 ± 6.4	80.1 ± 7.8	75.9 ± 5.3	78.8 ± 6.2	81.0	90.0 ± 2.8	79.3 ± 5.3	79.1 ± 6.8
L SLS R45 (%)	45.9 ± 2.9	44.7 ± 2.4	48.7 ± 2.6	44.4 ± 2.7	47.8 ± 2.3	44.8 ± 2.5	45.3 ± 3.0	45.3 ± 3.6	44.5 ± 3.1	45.9 ± 3.8	48.5	48.3 ± 2.0	45.7 ± 2.9	43.9 ± 3.1
L SLS L45 (cm)	71.0 ± 6.0	65.7 ± 6.5	75.0 ± 1.6	65.5 ± 9.3	67.1 ± 5.3	64.1 ± 6.2	69.1 ± 6.7	69.2 ± 7.6	68.5 ± 7.2	67.4 ± 8.5	78.0	77.0 ± 4.2	68.0 ± 5.7	67.0 ± 9.6
L SLS L45 (%)	40.6 ± 3.2	37.7 ± 3.4	42.0 ± 1.5	38.4 ± 5.4	40.4 ± 3.6	37.2 ± 3.6	40.2 ± 3.1	39.2 ± 3.9	40.1 ± 3.0	39.4 ± 5.4	46.7	41.5 ± 5.3	39.2 ± 3.6	37.1 ± 4.7
L SLS L135 (cm)	87.4 ± 9.7	87.4 ± 7.0	91.3 ± 9.4	75.2 ± 12.1	92.2 ± 6.5	79.5 ± 13.5	88.1 ± 8.9	91.1 ± 10.0	90.1 ± 4.4	86.2 ± 7.0	90.0	102 ± 2.8	87.8 ± 5.7	83.5 ± 11.3
L SLS R135 (cm)	65.9 ± 13.2	61.8 ± 13.9	63.3 ± 10.9	51.0 ± 12.2	68.8 ± 8.2	59.2 ± 12.0	65.5 ± 11.9	67.7 ± 9.8	63.3 ± 10.1	60.8 ± 12.7	66.0	72.0 ± 14.1	63.3 ± 11.0	55.0 ± 15.0
L SLS LROT (°)	133.3 ± 14.9	126.2 ± 16.2	135.0 ± 12.3	107.8 ± 25.7	127.6 ± 10.7	127.3 ± 16.5	131.9 ± 18.9	138.4 ± 12.6	131.4 ± 13.3	132.8 ± 17.6	150.0	122.0 ± 18.4	118.3 ± 11.6	128.2 ± 17.3
L SLS RROT (°)	136.5 ± 10.4	138.9 ± 13.4	138.8 ± 13.4	123.3 ± 11.0	131.0 ± 11.7	131.1 ± 12.9	135.2 ± 13.9	135.2 ± 14.7	131.8 ± 15.7	139.8 ± 10.1	128.0	143.0 ± 18.4	123.9 ± 9.1	132.1 ± 14.6
R SLS L45 (cm)	81.8 ± 4.9	79.8 ± 4.9	85.8 ± 1.5	77.5 ± 6.8	78.5 ± 6.0	78.6 ± 5.9	78.6 ± 6.3	81.3 ± 6.8	75.5 ± 5.7	79.1 ± 6.1	83.0	87.0 ± 4.2	81.3 ± 4.8	79.6 ± 7.4
R SLS L45 (%)	46.8 ± 2.6	45.8 ± 2.5	48.0 ± 2.1	45.5 ± 4.2	47.1 ± 2.6	45.5 ± 2.4	45.7 ± 2.8	46.0 ± 2.8	44.3 ± 2.4	46.2 ± 3.5	49.7	46.7 ± 1.1	46.8 ± 3.2	44.2 ± 3.3
R SLS R45 (cm)	71.1 ± 6.1	66.2 ± 5.9	72.5 ± 1.3	63.510.0	67.5 ± 5.9	63.4 ± 7.0	68.2 ± 6.6	70.3 ± 7.1	67.0 ± 5.8	67.0 ± 8.4	73.0	79.5 ± 2.1	68.6 ± 6.4	65.6 ± 9.3
R SLS R45 (%)	40.7 ± 3.4	38.0 ± 2.7	40.6 ± 0.72	37.2 ± 5.4	38.0 ± 11.3	36.8 ± 4.4	39.6 ± 3.2	39.8 ± 3.7	39.3 ± 2.3	39.1 ± 5.1	43.7	42.8 ± 4.2	39.5 ± 4.3	36.4 ± 4.7
R SLS R135 (cm)	86.5 ± 9.4	85.2 ± 12.9	91.0 ± 8.5	75.3 ± 9.2	92.8 ± 6.5	81.2 ± 12.4	84.7 ± 8.4	90.2 ± 8.3	89.1 ± 6.0	83.5 ± 7.1	83.0	104.0 ± 0.0	87.3 ± 5.9	80.8 ± 12.5
R SLS L135 (cm)	69.3 ± 12.8	66.2 ± 17.1	65.3 ± 10.4	49.7 ± 11.2	68.9 ± 8.8	60.9 ± 12.0	66.8 ± 10.3	68.6 ± 10.3	69.6 ± 10.3	60.4 ± 12.9	59.0	67.5 ± 17.7	62.5 ± 11.7	65.6 ± 9.3
R SLS RROT (°)	132.5 ± 17.8	127.4 ± 16.0	129.0 ± 22.8	107.0 ± 20.1	126.1 ± 10.1	123.9 ± 16.3	132.6 ± 15.3	136.8 ± 13.7	126.4 ± 18.9	130.4 ± 11.4	135.0	125.0 ± 14.1	115.92 ± 11.5	126.5 ± 17.4
R SLS LROT (°)	136.6 ± 12.2	135.9 ± 20.4	146.8 ± 18.1	124.0 ± 14.3	131.7 ± 8.5	132.0 ± 15.1	134.9 ± 14.4	136.5 ± 9.8	137.6 ± 14.6	138.1 ± 16.7	130.0	128.5 ± 5.0	124.2 ± 7.8	132.1 ± 14.6

*Values are means ± standard deviations; CC, Cross country skiing; R, Right; L, Left; 45, 45 degree relative to anterior surface of body; 135, 135 degrees relative to anterior surface of body: ROT, Rotation; %, normalized HSEBT values (reach measurement/wingspan·100)*.

**Table 2:** Age, anthropometry, and HSEBT results (absolute and normalized values) grouped by gender, age, and activity level with group comparisons.

	**Male**	**Female**	***d***	***p***	**Young**	**Adult**	***d***	***p***	**Athletes**	**Recreational**	***d***	***p***	**MDC**
*n*	133	90			159	64			166	57			
Age (yrs)	20.2 ± 4.3	17.6 ± 1.8	0.83	<0.001	17.1 ± 0.6	24.3 ± 3.4	12.5	<0.001	17.4 ± 1.4	24.1 ± 3.9	2.00	<0.001	
Height (cm)	177.5 ± 7.1	167.1 ± 6.7	1.51	<0.001	170.6 ± 8.0	179.6 ± 7.0	1.17	<0.001	170.7 ± 7.9	180.4 ± 6.8	1.26	<0.001	
Leg length (cm)	90.3 ± 4.6	86.1 ± 4.5	0.94	<0.001	87.5 ± 4.6	91.2 ± 5.0	0.78	<0.001	87.7 ± 4.7	91.3 ± 4.4	0.79	<0.001	
Wingspan (cm)	179.4 ± 8.2	168.5 ± 7.9	1.35	<0.001	173.0 ± 9.6	179.4 ± 8.5	0.68	<0.001	173.0 ± 9.6	180.2 ± 8.1	0.77	<0.001	
Arm length (cm)	75.0 ± 3.3	70.3 ± 3.6	1.38	<0.001	72.4 ± 4.3	74.7 ± 3.7	0.56	<0.001	72.4 ± 4.3	75.0 ± 3.4	0.64	<0.001	
R45 (cm)	80.1 ± 6.4	78.4 ± 5.8	0.28	0.043	79.3 ± 6.0	79.4 ± 6.3	0.01	0.942	79.4 ± 5.9	79.4 ± 6.9	0.01	0.928	1.5–2.1
R45 NORM (%)	44.7 ± 2.9	46.5 ± 2.8	0.64	<0.001	45.9 ± 2.8	44.4 ± 3.2	0.50	0.001	45.9 ± 2.8	44.0 ± 3.1	0.66	<0.001	NE
L45 (cm)	68.1 ± 7.6	68.4 ± 6.0	0.04	0.760	68.3 ± 6.8	67.7 ± 7.8	0.08	0.583	68.4 ± 6.7	67.5 ± 7.8	0.13	0.396	2.4–2.8
L45 NORM (%)	38.0 ± 3.9	40.6 ± 3.1	0.72	<0.001	39.6 ± 3.5	37.7 ± 4.0	0.50	0.001	39.7 ± 3.5	37.2 ± 3.9	0.67	<0.001	NE
L135 (cm)	85.6 ± 9.6	87.3 ± 7.8	0.19	0.181	87.0 ± 8.7	84.9 ± 8.7	0.24	0.110	87.5 ± 8.2	83.4 ± 9.6	0.48	0.003	3.9–4.2
R135 (cm)	60.7 ± 13.3	65.3 ± 9.7	0.41	0.004	64.5 ± 11.6	56.9 ± 14.6	0.55	<0.001	65.0 ± 11.1	54.5 ± 14.9	0.75	<0.001	7.2–7.9
LROT (°)	128.3 ± 16.1	129.8 ± 13.1	0.10	0.462	130.0 ± 13.7	125.5 ± 16.5	0.31	0.038	129.2 ± 14.0	127.3 ± 16.6	0.12	0.426	6.3–7.2
RROT (°)	134.4 ± 13.1	133.1 ± 10.5	0.11	0.431	134.8 ± 11.1	132.6 ± 14.0	0.17	0.211	134.9 ± 11.3	134.7 ± 14.1	0.06	0.679	4.7–5.2

*Values are means ± standard deviations; R, Right; L, Left; 45, 45 degree relative to anterior surface of body; 135, 135 degrees relative to anterior surface of body; ROT, Rotation; %, normalized HSEBT values (reach measurement/wingspan·100); MDC, Minimal detectable change; NE, Not established*.

### Regression Analysis

Multicollinearity (VIF ranged from 1.000-4.152) was not observed. Homogeneity of variance was observed, with residuals being independent (Durbin-Watson ranged from 1.699-2.397). Wingspan explained 34.6 and 11.7% of the variance in the R45 and L45 reach measurements, respectively. Leg length explained 2.7% of the L135 reach (Table 3). No anthropometric variable could explain a significant portion of the variance in the R135, LROT, and RROT reaches. Based on the aforementioned criteria, only the L45 and R45 measurements were normalized to wingspan and expressed as a percentage of wingspan. In R45 and L45 reaches, sex and leg length had a non-significant contribution in the validation model (Table A1). In addition, activity level and age explained 3.3 and 6.5%% of the L135 and R135 reaches, respectively (Table 3).

**Table 3:** Stepwise multiple linear regression of HSEBT reaches.

**Test**	***B***	**SE *B***	**B**	***R*^**2**^**
**R45 STEP 1**				
Constant	11.96	7.22		
Wingspan	0.39	0.041	0.59^***^	0.346
**R45 STEP 2**				
Constant	−3.93	8.45		
Wingspan	0.47	0.047	0.62^***^	
Sex	3.07	0.92	0.24^***^	0.388 (Δ R^2^ = 0.042)
**R45 STEP 3**				
Constant	0.62	8.58		
Wingspan	0.58	0.069	0.89^***^	
Sex	3.1	0.90	0.24^***^	
Leg length	−0.279	0.12	−0.22^*^	0.407 (Δ R^2^ = 0.019)
**L45 STEP 1**				
Constant	22.71	9.69		
Wingspan	0.26	0.055	0.34^***^	0.117
**L45 STEP 2**				
Constant	−3.86	11.13		
Wingspan	0.40	0.062	0.53^***^	
Sex	5.15	1.21	0.35^***^	0.206 (Δ R^2^ = 0.089)
**L135 STEP 1**				
Constant	87.38	0.83		
Activity level	−3.86	1.67	−0.18^*^	0.033
**L135 STEP 2**				
Constant	59.67	12.86		
Activity level	−4.64	1.69	−22^**^	
Leg length	0.32	0.15	0.17^*^	0.060 (Δ R^2^ = 0.027)
**R135**				
Constant	64.68	1.08		
Activity level	−7.21	2.17	−0.25^**^	0.065
**RROT**				
NE
**LROT**				
NE

* p < 0.05

** p < 0.01

*** p < 0.001

*B, Unstandardized coefficient; β, Standardized beta coefficient; SE, Standard error; R^2^, Coefficient of determination; NE, No variables entered into the equation; R = Right; L = Left; 45, 45 degree relative to anterior surface of body; 135, 135 degrees relative to anterior surface of body: ROT, Rotation*.

## Discussion

### Influence of Anthropometry

Anthropometric measures influence HSEBT reach measurements differently, therefore reach specific normalization should be used. Flexion movement patterns yielded expected results: wingspan explained 11.7 and 34.6% of the variation in reach measurements. In addition, normalizing flexion movement patterns to wingspan resulted in significant differences for all groups (age, sex, and activity level). However, extension movement patterns unexpectedly were not influenced by any of the anthropometric measures. Leg length did explain 2.7% of the variation in the R135 reach measurement. However, this is less than the previously established CV (Eriksrud et al., 2017). In addition, leg length did not significantly correlate with the R135 reach measurement, suggesting normalization to leg length is not needed. As expected, the rotational reaches do not require normalization. The reach specific considerations for HSEBT normalization differ from the normalization procedures proposed by Gribble and co-workers for the SEBT (Gribble and Hertel, 2003). In their study leg length was found to have greater coefficients of determination than height to SEBT reaches (0.02–0.23), with significant correlations in six of eight SEBT reach measurements (Gribble and Hertel, 2003). Although lateral and posterolateral reaches were not significantly correlated with leg length, all SEBT reaches are normalized to this measure and since then widely applied (Gribble et al., 2012). In fact, leg length explained 4% of the variance of the posterolateral reach measurement (Gribble and Hertel, 2003), which is less than the CV for test-retest reliability (4.4%) (Plisky et al., 2006). The normalization of HSEBT measurements to anthropometric variables which explain variation beyond error, as done in the current study, appears to be a more appropriate procedure.

### Influence of Age

There appears to be an effect of age on HSEBT reach measurements. Specifically, the young group has greater measurements in three of six reaches. Medium effects of age were observed for the normalized L45 and R45 reaches, as well as for the R135 reach. However, the group difference observed for the R135 reach (7.6 cm) is within the range of MDC values (Table 2). In their study Eriksrud and co-workers recommend 7 cm as an MDC for extension movement patterns based on calculations and clinical experience (Eriksrud et al., 2017). It is important to note that the MDC values in this study were calculated based on a 95% confidence interval, which is more conservative and generate greater values than the 90% confidence interval commonly used (Haley and Fragala-Pinkham, 2006). Consequently, we interpreted from our findings that the young participants had greater R135 reach measurements. The combination of significant group differences, effect sizes and comparison to established MDC values (R135) allows for a more robust interpretation of our findings. However, age did not explain a significant portion of the variation of any of the reach measurements in the regression analysis. Thus, it appears that age should be considered cautiously when performing between individual or group comparisons for the normalized L45 and R45 as well as for the R135 reach.

These findings contradict the influence of age on other measures of dynamic postural control such as the SEBT, where reach measurements increase with age (Holden et al., 2016; Gonzalo-Skok et al., 2017; McCann et al., 2017). However, these findings are based on young populations. Older basketball players (16 years) had increased SEBT measurements in some directions when compared to younger players (14 years) (Gonzalo-Skok et al., 2017). In a similar age group Holden and co-workers reported that 13-year-olds increased all SEBT reaches tested over a 24-months period (Holden et al., 2016), while McCann and co-authors reported that older (20 years) had greater SEBT reach measures than younger (15 years) football players (McCann et al., 2017). However, only one study reported effect sizes (Gonzalo-Skok et al., 2017), and these studies did not compare group differences to recommended MDC values (5–7cm; 6–8% of leg length) (Munro and Herrington, 2010). Comparisons to these MDC values would change the interpretation of findings in the aforementioned studies. Older basketball players would still have greater SEBT reaches (Gonzalo-Skok et al., 2017) whereas older football players would not (McCann et al., 2017) in comparison to their younger counterparts. In addition, the observed increase in SEBT reaches over a 24-months period would only apply to the posterolateral reach (Holden et al., 2016). In the current study we calculated not only if group differences were significant, but also effect sizes before determining if group differences were greater than MDC values. This is a more robust analysis in comparison to what has been done for the SEBT, and allows us to be more certain about the effect of age on HSEBT reaches.

### Influence Activity Level

Athletes have greater HSEBT reach measurements than recreationally active for three of six reaches. These reaches are the same as for the age group comparisons: normalized L45 and R45 reaches as well as the R135 reach. These group comparisons had medium effects, and the group difference for the R135 reach was greater than MDC values. Furthermore, activity level explained 3.3 and 6.5% of the variance of the L135 and R135 reaches. However, these values are less than most of the observed CV's for these reaches (5.2–14.6%) (Eriksrud et al., 2017). In addition, the observed influence of activity level on these HSEBT reaches are influenced by age, since the athlete group was significantly younger than the recreational group (large effect) (Table 2). Based on these findings, activity level should be considered when performing between individual or group comparisons for the normalized L45 and R45 as well as the R135 reach.

The influence of activity level on SEBT reaches has been found to be equivocal. Specifically, female modern dancers have better reach performance in some, but not all reach directions, in comparison to active non-dancers (Ambegaonkar et al., 2013). In a study comparing basketball players Sabin and co-authors found that active controls had greater SEBT reach measurements than basketball players (Sabin et al., 2010). Thorpe and co-authors found that female soccer players (NCAA division 1) had greater SEBT reach measurements than their recreationally active counterparts (Thorpe and Ebersole, 2008). In addition, there are SEBT reach differences between athletes participating in different sports. Specifically, soccer players have greater SEBT reaches than basketball players, while there is no difference between gymnasts and soccer players (Bressel et al., 2007). However, these studies neither report effect sizes nor compare to MDC values as advocated by Munro and co-authors (Munro and Herrington, 2010). Comparing group differences to MDC values in the aforementioned studies influence interpretation of findings. Specifically, dancers would not have demonstrated greater SEBT reaches than non-dancers (Ambegaonkar et al., 2013), and basketball players would only have lower SEBT measurements in the anterior direction, and not in the medial and posterior (Sabin et al., 2010). Furthermore, soccer players would still have greater anterior and posterior reaches than their active controls (Thorpe and Ebersole, 2008). Overall, these findings indicate that there might not only be activity but also sports specific adaptations of dynamic postural as measured by the SEBT. In the current study it was not possible to determine sport specific adaptations due to the small sample sizes of the different sports included (Table 1), but the influence of activity level (athletic vs. recreational participation) could be analyzed. Since we calculated effect sizes and compared the group difference to established MDC values (R135), the inference that activity level leads to greater L45, R45, and R135 measurements is justified. However, some caution should be applied to the interpretation of these findings considering the that the athletic population was significantly younger (large effect), and that a smaller percentage of the reach measurement variance (3.3–6.5%) of only the L135 and R135 reaches could be explained by activity level.

### Influence of Sex

Females had significantly greater HSEBT reach measurements for normalized L45 and R45 reaches with a medium effect. These findings could be influenced by the female group being younger than the male group (*d* = 0.83) since younger participants have greater normalized L45 and R45 reach measurements as discussed previously. It is interesting to note that males have significantly greater absolute R45 reach measurements with a small effect and a group difference less than MDC values. Normalization to wingspan changes this relationship completely with females having greater measurements (*d* = 0.64). These findings might be due to males having a greater wingspan (10.9 cm; *d* = 1.51), and that the R45 reach is where wingspan accounts for the greatest variation of the measurement (34.6%). Thus, females are better able to combine different joint movements to maximize R45 reach measurements despite having unfavorable anthropometrics.

Similar to our findings physically active females have been found to have greater SEBT reach measures than their male counterparts (Gribble et al., 2009). However, in their study Gribble and co-authors found no influence of sex on normalized SEBT reach measurements, and males having greater absolute SEBT reach measurements (Gribble and Hertel, 2003). Contrary to our findings, others have found males (Sabin et al., 2010) and athletic males (Gorman et al., 2012b) to have greater SEBT measures than their female counterparts. In the aforementioned studies neither effect sizes were reported nor were group differences compared to MDC values (Munro and Herrington, 2010). The group differences presented by Gribble and co-authors (Gribble and Hertel, 2003) are less than the established MDC values except for the posterior reach, while the group differences presented by Gribble and co-workers in their later study (Gribble et al., 2009) were all lower than established MDC values (visual interpretation from graphs). The values presented by Gorman and co-authors cannot be compared to MDC values since it is impossible to extract them from the graphs presented (Gorman et al., 2012b). Thus, it appears that sex has a small influence on SEBT reach measurements. Since sex had a medium effect and explained 4.2 and 8.9% of the variance of the R45 and L45 reach measurements respectively, greater than most CV's for R45 and L45 reaches (3.0–5.2%) (Eriksrud et al., 2017), it appears that sex influence these HSEBT reaches. However, sex was not found to have a significant contribution to the validation model for the R45 and L45 reaches. Thus, the interpretation of sex influencing these reaches should be done cautiously.

### Outlook, Clinical Implications and Limitations

The current study established that HSEBT flexion movement patterns should be normalized to wingspan. However, wingspan explains only 34.6 and 11.7% of the variation in R45 and L45 reach measurements, respectively. This leaves a large percentage of the variance to be determined by other factors. To date the HSEBT has been proven to be reliable and valid (Eriksrud et al., 2017) and measuring different aspects of dynamic postural control than the SEBT (Eriksrud et al., 2018). SEBT reaches have been found to reflect different neuromuscular functions such as proprioception (Belley et al., 2016), lower extremity strength (Hubbard et al., 2007; Crossley et al., 2011; Norris and Trudelle-Jackson, 2011), muscular power (Booysen et al., 2015), and balance (Hubbard et al., 2007). A better understanding of the influence of neuromuscular functions on HSEBT reach measurements should be explored.

The current study has shown that age, sex and activity level influence HSEBT measurements and consequently should be considered when performing between individual and group comparisons. The age groups compared in the current study were teenagers (age 17.1 ± 0.6) and young adults (age 24.3 ± 3.4). To better understand the influence of age on the HSEBT, larger age ranges (>10 years) should be tested with measurements organized in age groups, as done for ROM data (Bell and Hoshizaki, 1981). This will allow for the development of reference values and the exploration of how HSEBT reach develops the across the life span. The development of such reference values can be important. Specifically, in an older population they can be useful in fall risk management, since the HSEBT is situation specific to risky movements such as reaching, leaning (Nachreiner et al., 2007) and bending (Duckham et al., 2013).

The HSEBT can be used to measure sports and activity dependent adaptations and characteristics and their influence on performance. In the current study, due to small sport specific sample sizes, we could only explore the influence of activity level and not sport specific adaptations and characteristics. Even if between sport comparisons were not done, we have presented reference data for different winter sports for future comparisons. The authors expect that athletes participating in different sports will have different HSEBT reach capacities. Specifically, sports where the use of the upper extremities is fundamental to the activity (golf, tennis, volleyball, overhead throwing sports etc.) are expected to show greater reach measures as compared to sports where the upper extremities are less important (i.e., soccer). In addition, specific cut-off values for athletic performances can be determined. For instance, it might be that extension movement pattern measurements up to a certain value increase tennis serve speed, while a further increase does not. Such reference and performance specific cut-off values can be useful in the development and rehabilitation of athletes.

## Conclusion

Flexion movement patterns (L45 and R45 reaches) should be normalized to wingspan, since a significant variation of these measurements is explained by this measure. In fact, only when normalized L45 and R45 reach measurements were compared, group differences for age, sex and activity level became significant. On the contrary, extension movement patterns do not need to be normalized to anthropometric measures since only leg length had a small influence on the L135 reach measurement. Neither anthropometric measures nor age, sex and activity level influence the rotational reaches. Thus, reference and predictive values for research and clinical purposes should be based on flexion movement patterns normalized to wingspan. In a young and adult population it appears that age, sex and activity level influence HSEBT reach measurements.

## Ethics Statement

Informed consent was obtained by electronic means and data was stored anonymously. The study was authorized and approved by Regional Committees for Medical and Health Research Ethics and the National Data Protection Authority and conducted according to the Declaration of Helsinki.

## Author Contributions

OE contributed to conception of the idea and design of the study. All authors OE, PF, and JC contributed to data analysis and writing of the manuscript.

### Conflict of Interest Statement

OE is a co-founder of Athletic Knowledge AB (Stockholm, Sweden) which commercially distributes a testing mat for HSEBT and SEBT. The remaining authors declare that the research was conducted in the absence of any commercial or financial relationships that could be construed as a potential conflict of interest.

## Appendix

**Table A1:** Validation of the multiple linear regression of HSEBT reaches.

**Initial model (75%)**					**Validation model (25%)**			
**Test**	***B***	**SE *B***	**B**	***R*^2^**	***B***	**SE *B***	**B**	***R*^2^**
**R45 STEP 1**								
Constant	11.96	7.22						
Wingspan	0.39	0.041	0.59^***^	0.346				
**R45 STEP 2**								
Constant	–3.93	8.45						
Wingspan	0.47	0.047	0.62^***^					
Sex	3.07	0.92	0.24^***^	0.388 (ΔR^2^ = 0.042)				
**R45 STEP 3**					**FORCED ENTRY**			
Constant	0.62	8.58			13.85	14.944		
Wingspan	0.58	0.069	0.89^***^		0.30	0.14	0.52^*^	
Sex	3.1	0.90	0.24^***^		2.27	1.73	0.22	
Leg length	–0.279	0.12	–0.22^*^	0.407 (ΔR^2^ = 0.019)	0.135	0.242	0.12	0.288
**L45 STEP 1**								
Constant	22.71	9.69						
Wingspan	0.26	0.055	0.34^***^	0.117				
**L45 STEP 2**					**FORCED ENTRY**			
Constant	–3.86	11.13			13.00	17.11		
Wingspan	0.40	0.062	0.53^***^		0.32	0.095	0.50^**^	
Sex	5.15	1.21	0.35^***^	0.206 (ΔR^2^ = 0.089)	0.93	2.02	0.068	0.215
**L135 STEP 1**								
Constant	87,38	0.83						
Activity level	–3.86	1.67	–0.18^*^	0.033				
**L135 STEP 2**					**FORCED ENTRY**			
Constant	59.67	12.86			44.16	19.64		
Activity level	–4.64	1.69	–22^**^		–7.35	2.71	–0.42^**^	
Leg length	0.32	0.15	0.17^*^	0.060 (ΔR^2^ = 0.027)	0.49	0.22	0.34^*^	0.134
**R135**					**FORCED ENTRY**			
Constant	64.68	1.08			66.07	1.69		
Activity level	–7.21	2.17	–0.25^**^	0.065	–14.20	3.17	–0.53^***^	0.28
**RROT**					**FORCED ENTRY**			
NE					NE			
**LROT**					**FORCED ENTRY**			
NE					NE			

* p < 0.05

** p < 0.01

*** p < 0.001

B, Unstandardized coefficient; β, Standardized beta coefficient; SE, Standard error; R^2^, Coefficient of determination; NE, No variables entered into the equation; R, Right; L, Left; 45, 45 degree relative to anterior surface of body; 135, 135 degrees relative to anterior surface of body: ROT, Rotation.
